# Biological Activities and Chemistry of Triterpene Saponins from *Medicago* Species: An Update Review

**DOI:** 10.1155/2021/6617916

**Published:** 2021-05-26

**Authors:** Guanzhen Wang, Junlong Wang, Wei Liu, Muhammad Farrukh Nisar, Mohamed A. El-Esawi, Chunpeng Wan

**Affiliations:** ^1^Key Lab of Natural Product Chemistry and Application at Universities of Education Department of Xinjiang Uygur Autonomous Region, Yili Normal University, Yining 835000, China; ^2^College of Agronomy, Jiangxi Agricultural University, Jiangxi Key Laboratory for Post-harvest Technology and Nondestructive Testing of Fruits & Vegetables/Collaborative Innovation Center of Post-harvest Key Technology and Quality Safety of Fruits and Vegetables in Jiangxi Province, Nanchang 330045, China; ^3^Key Laboratory of Crop Physiology, Ecology and Genetic Breeding, Ministry of Education, Jiangxi Agricultural University, Nanchang, Jiangxi, China; ^4^Department of Physiology and Biochemistry, Cholistan University of Veterinary and Animal Sciences (CUVAS), Bahawalpur 63100, Pakistan; ^5^Botany Department, Faculty of Science, Tanta University, Tanta 31527, Egypt

## Abstract

Plants are known to be a great source of phytochemicals for centuries. *Medicago*, belonging to the Family Fabaceae, is a large and well spread genus comprising about 83 cosmopolitan species, of which one-third are annuals and span diverse ecological niches. *Medicago* species are rich in saponins mainly classified into three classes, namely, steroid alkaloid glycosides, triterpene glycosides, and steroid glycosides. These saponins are important compounds having diverse pharmacological and biological activities. As a whole, 95 of saponins are reported to date occurring in *Medicago* species using various latest extraction/isolation techniques. Considering the multiple biological and pharmacological potential of *Medicago* species due to saponins along with structural diversity, we compiled this review article to sum up the recent reports for the pharmacological potential of the *Medicago*'s derived saponins in modern as well as traditional medication systems. The current manuscript produces data of chemical structures and molecular masses of all *Medicago* species saponins simultaneously. The toxicity of certain pure saponins (aglycones) has been reported *in vitro*; hederagenin appeared highly toxic in comparison to medicagenic acid and bayogenin against X. *index*, while soyasaponin I, containing soyasapogenol B as a glycone, appeared as the least toxic saponin. The diversity in the structural forms shows a close relationship for its biological and pharmacological actions. Moreover, saponins showed antioxidant properties and the mechanism behind antimicrobial potential also elaborated in this review article is mainly because of the side sugar groups on these compounds. The collected data presented herein include chemical structures and molecular masses of all saponins so far. Their biological activity and therapeutic potential are also discussed. This information can be the starting point for future research on this important genus.

## 1. Introduction

Plants are well known for huge source of diverse array of phytochemicals. Flowering plant family Fabaceae is the largest and well spread family throughout the world, and *Medicago* is one of its important genera comprising about 83 well-spread species, of which one-third are annuals [1]. The plants of the genus *Medicago* span diverse environmental conditions and are rich in alkaloids, flavonoids, naphthoquinones, and saponins [2–4]. Saponins comprise a huge range of glycosidic secondary metabolites reported in the genus *Medicago* [5]. Saponins are actually non-protein amino acids. Complexes of polysaccharides and proteins such as lectins and enzyme inhibitors are also included in the saponins that behave as plant protectors. The genus *Medicago* is reported to possess higher levels of saponins, and these are named due to their foam-forming properties. In plants, saponins mainly occur in *Medicago* species classified into three classes, namely, steroid alkaloid glycosides, triterpene glycosides, and steroid glycosides [6].

Different *Medicago* species are reported to contain different kinds and variable amounts of saponins. Structurally diverse class of saponins may contain mono-, bi-, or tridesmosidic, linear or either branched by linking with the aglycone moiety (sapogenin) through an ether of ester bond. This structural diversity in saponins shows a huge range of hydrophobicity, acidity, and polarity which defines their multiple pharmacological as well as biological actions [6]. Many *Medicago* plant species are toxic for herbivores due to their higher saponins quantity, but *Medicago* saponins are reported to be toxic for birds and animals [7, 8].

Different parts, namely, roots, stem, leaves, and seeds, have different concentrations of various saponins. Keilbasa and colleagues extracted saponins from *M. sativa* leaves, roots, and sprouts and reported highest concentration of sapogenins in the roots, then sprouts and the least amounts in the leaves [9]. Moreover, it is further stressed that the extraction solvent and analytical technique may define the exact amounts of particular saponins in specific part of the *Medicago* plants. The edaphic factors of the *Medicago* plants also impart the production of specific saponins at all levels including the quantity [10]. Moreover, if the roots of *M. truncatula* species develop symbiotic relationship with certain mycorrhizal fungi, the concentration and amount of secondary metabolites including triterpene saponins also increase in *M. truncatula* plants having mycorrhizal association than those plants without mycorrhizal association [11]. In certain molecular engineering studies, the modulation of *β*-amyrin synthase-encoding sequence (AsOXA1) in *M. truncatula* showed raised biosynthesis of triterpene saponins and nodulation in roots [12]. Two *M. truncatula* cytochromes P450 enzymes (MtCYP72A67 and MtCYP72A68) along with *β*-amyrin synthase, MtCYP716A12 are involved in biosynthesis of saponins, and it was found that more saponins were produced due to the expression of these genes [13].

Among various natural phytochemicals, *Medicago* saponins are of great interest due to their wide spectrum pharmacological and biological properties [2]. These saponins in *Medicago* species are being formed when large molecules of triterpene glycosides get complexed with zahnic acid, hederagenin, medicagenic acid, bayogenin, and soyasapogenols A and B as the leading aglycones [14, 15]. These *Medicago* saponins are highly effective against tumorigenesis, fungal growth, and have cytotoxic effects for mollusks, bacteria, and various viruses [6, 16, 17].

Keeping in view the diverse biological and pharmacological activities of saponins along with structural diversity, the most recent available literature about the saponins has been reviewed. Moreover, the nematicidal potential of saponins found in various *Medicago* species along with antioxidant properties shall also be discussed using latest literature to give an update of this important class of compounds. The collected data presented herein includes chemical structures and molecular masses of all saponins so far. Their biological activity and therapeutic potential are also discussed. This information can be the starting point for future research on this important genus.

## 2. Extraction, Separation, Identification, and Quantification of Saponins

Saponins are secondary plant metabolites distributed in the plant kingdom in several species, and they encompass triterpenoids, steroids, and steroidal alkaloids glycosylated having single or multiple sugar residues or chains [18]. Contents and composition profile of saponins depend on the cultivar, environmental conditions, physiological stage of growth, and plant organ. The saponin amount varied according to the species, ranging from 0.38 ± 0.04% for *M. rugosa* Desrouss. to 1.35 ± 0.08% for *M. scutellate* (L.) Mill. Medicagenic acid was the dominant aglycone in *M. blancheana*, *M. doliata*, *M. littoralis*, *M. rotata*, *M. rugosa*, *M. scutellata*, *M. tornata*, and *M. truncatula*, echinocystic acid in *M. polymorpha*, hederagenin and bayogenin in *M. rigidula* and *M. arabica*, and soyasapogenol B in *M. aculeata* [19]. The pharmaceutical property discoveries from the *Medicago* species have driven the emergence of various extraction technologies with the main purpose of maximizing the yield in order to accommodate the recent need. Therefore, Cheok et al. reviewed the extraction and quantification of saponins [20]. In general, the extraction techniques employed in saponin extraction are Soxhlet, maceration, and reflux extraction, microwave-assisted, ultrasound-assisted, and accelerated solvent extraction. The quantification of plant saponins is usually carried out by UV-spectrophotometric and chromatographic (HPLC, UPLC, TLC) methods [19]. Saponins are separated and purified from plant materials using chromatographic methods in many studies to identify a specific saponins compound and investigate its pharmaceutical property [20]. Sapogenins are usually obtained after acid hydrolysis of saponins and evaluated by GC/FID and GC/MS methods [19]. The elucidation and characterization of saponins structure are conducted usually on the basis of EI-MS, 1D, and 2D NMR data [20].

## 3. Chemical Constituents

**Figure 1:**
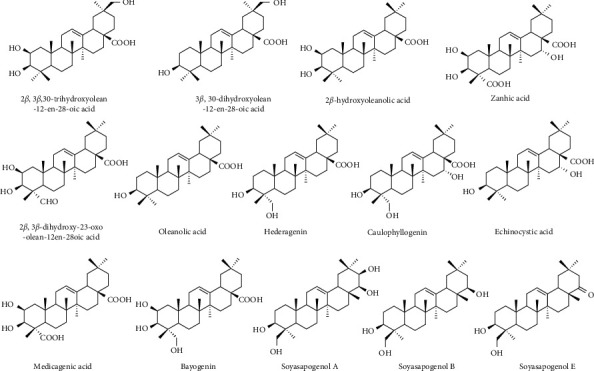
Chemical structure of sapogenins detected in *Medicago* species.

For the genus *Medicago*, saponins make highly complex blend of glycosidic triterpenes originally derived from the isoprenoid pathway via the cyclization of 2,3-oxidosqualene to form *β*-amyrin nucleus. Oxidative modifications are driven by a series of cytochromes P450 (CYPs) and generate the aglycone moieties (sapogenins) that are subjected to glycosyl transfer reactions mediated by glycosyltransferases to give the different saponins. On this basis, 2 groups of saponins are reported in the *M. sativa* that can be differentiated: (1) sapogenins with COOH at the C-28 and different oxidation states (zero, OH, CHO, COOH) at C-23 (medicagenic acid, zanhic acid, hederagenin, bayogenin, 2*β*, 3*β*-dihydroxy-23-oxo-olean-12-en-28-oic acid); and (2) sapogenins with an OH group at C-24 with no substituent at C-28 (soyasapogenol A, B, E) [21]. Recently the queretaroic acid and its 2 *β*-hydroxy derivative, 2*β*, 3*β*, 30-trihydroxyolean-12-en-28-oic acid have been identified as novel aglycon in *M. arabica* (Figure 1). Queretaroic acid has the olean-12-ene skeleton and, together with glycyrrhetic acid, is one of the few naturally occurring triterpenes which is oxygenated at C-30 [22]. Queretaroic acid is supposed to be synthesized *in vivo* by a CYP P450 dependent hydroxylation of oleanolic acid [23].

In-depth examinations are conducted to elucidate chemical structures of saponins (compounds 1–95) in *M. arabica, M. marina, M. polymorpha, M. truncatula, M. sativa,* and *M. arborea*. Various saponins characterized till now from these species of *Medicago* are described in Tables 1–6. Various above-ground parts of *M. arabica* are well characterized to report the occurrence of saponins comprising of short chain sugar residues such as mono and bidesmosides of hederagenin, bayogenin, 2*β*-hydroxy oleanolic acid, soyasapogenol B, and oleanolic acid. An exciting quality of saponins derived from *M. arabica* is bidesmosides of 3*β*, 30-dihydroxyolean-12-en-28-oic acid and 2*β*, 3*β*, 30-trihydroxyolean-12-en-28-oic acid (compounds 1–5), as new aglycons for saponins of *Medicago* species (Table 1). All the detected saponins in *M. marina* are bidesmosidic compounds with the C-3 position characterized by the presence of the same sugar, glucose or by the disaccharide chain Glc (1⟶2) Glc (Table 2). Compounds 20, 21, 25, and 26 are undescribed in *Medicago* and never reported before in other plant species. Twelve triterpene saponins are recognized as glycosides of echinocystic acid hederagenin, soyasapogenol B, bayogenin, and caulophyllogenin in *M. polymorpha* (Table 3). Compounds 31 and 32 are declared as the novel natural compounds in *Medicago* species. Echinocystic acid is pioneer compounds to be reported in the genus *Medicago*. Saponins in *M. truncatula* seeds consist mainly of mono- and bidesmosides of soyasapogenol B and medicagenic acid (Table 4). Thirty-five pentacyclic triterpenoid saponins in *M. sativa* have been reported to occur as a complex mixture of short and long sugar chains of mono and bidesmosidic compounds having zanhic acid, bayogenin, hederagenin, medicagenic acid, 2*β*, 3*β*-Dihydroxy-23-oxo-olean-12en-28oic acid and soyasapogenol B (Table 5). Compounds 62, 77, 78, 84–88, and 91 are new triterpenoid saponins, but methyl ester derivative of saponins (compounds 77, 78, and 88) are accepted as artifacts examined through methanolic extraction [24]. *M. arborea* saponins from aerial parts are mainly mono and bidesmosides of medicagenic acid (Table 6).

## 4. Biological Activity

Being the model plant species, *Medicago* holds a prominent place in Leguminosae family mainly due to its saponins [36]. The presence of diverse class of chemicals holding multiple biological activities is all well reported and utilized for centuries. These saponins are primarily the glycosides having aglycone moiety which is formed involving enzymatic cyclization of 2,3-oxidosqualene catalyzed by the *β*-amyrin cyclase [22]. Most of the *Medicago* species are being utilized as fodder for the grazing animals, but traditional medication system also clarifies that some of the species such as *M. sativa* herb are also beneficial for the human body. *M. sativa* is well recognized for centuries in traditional medication system in curing loss of memory, kidney issues, asthma, coughing, joint pains, and central nervous system disorders. All these pharmacological activities are detailed in the following text.

### 4.1. Insecticidal Activity

Due to increasing environmental and public health issues of using synthetic pesticides, the scientists are ever trying their hard to explore safer biological molecules to cure agricultural crops against multiple pathogens, namely, insects, bacterial, and fungal strains. Plant parasitic nematodes are cosmopolitan in distribution and are a major cause of huge economic losses for most of the agricultural crops and often quite hard to control the pathogens [37]. *M. sativa* L. shoot contains large amounts of saponins, which were identified in a recent study for their biological against aphid feeding, and found strong aphid inhibitory effects [38]. In an *in vitro* study, saponin rich mixtures of *M. sativa* showed effective growth inhibition on the viral vector nematodes like *Xiphinema*, the root-knot nematode *Meloidogyne incognita*, and *Globodera rostochiensis* which are the potato cyst parasites [37]. Three saponins, namely, 3-O-[*β*-D-glucuronopyranosyl]-28-O-[*α*-L-rhamnopyranosyl(1⟶2)-*α*-L-arabinopyranosyl] medicagenic acid, Zanhic acid tridesmoside and 3-O-[*β*-D-glucuronopyranosyl]-28-O- [*β*-D-xylopyranosyl (1⟶4)-*α*-L-rhamnopyranosyl (1⟶2)-*α*-L-arabinopyranosyl] medicagenic acid were extracted from *M. sativa* L., potentially inhibits feeding of aphid *Acyrthosiphon pisum* assessed through electrical penetration graph technique in a dose dependent way [38]. In another recent study, saponins (10, 100 ppm) extracted from *M. sativa* extracts were applied freshly ecdysed 3^rd^ larval instar of *Spodoptera littoralis*, and higher dose (100 ppm) caused absolute death while lower dose (10 ppm) caused only 26.7% mortalities [30]. Saponins mainly exert their effects by decreasing viability and rising mortalities, lowering the weights, reducing development and reproductive activities. Moreover, *M. sativa* saponins damaged the hindgut and fat body of *S. littoralis* badly to reduce its populations [30]. Another study examined the nematicidal effects of saponins of three different *Medicago* species (*M. sativa*, *M. arabica*, *M. arborea*) using plant shoots and roots against *Xiphinema index*, which is a plant parasitic nematode. It is said that the presence of prosapogenins and sapogenins in shoots and roots extracts (500 *μ*g/ml) effectively induces absolute (100%) mortality of *X. index,* except the *M. arborea* that is less effective within 48 hours [39]. This nematicidal activity is correlated with the presence of aglycones (medicagenic acid and hederagenin) that occur in the roots and shoots saponin extracts [40]. *M. truncatula* saponins mediate caterpillar deterrence as a resistance mechanism in F83005.5 ecotype and associate these saponins as potential antifeedants that could be used in agricultural sustainable pest management strategies.

The seeds flour of *M. truncatula* showed a strong inhibition of the major pest (rice weevil *Sitophilus oryzae*) of cereals including rice [28], which were mainly responsible to the constituent of saponins 3-GlcA-28-AraRhaxylmedicagenate. Furthermore, when the saponin 3-GlcA-28-AraRhaxylmedicagenate was used in less concentration, it showed no effects on *Caenorhabditis elegans* (*C. elegans*) and *E. coli*, but at higher concentrations (100 *μ*g/ml) it may lead to stopping the growth of *Saccharomyces cerevisiae*. Continuing this, the study emphasized the use of this target specific saponin (3-GlcA-28-AraRhaxylmedicagenate) only for mature *S. oryzae* but not others like coleopteran *Tribolium castaneum* and the Sf9 insect cultured cells [28]. Root knot nematodes *Meloidogyne incognita* is the major cause of huge economic losses and is quiet hard to control. *M. sativa* L. crude extracts are much effective against tomato seedling infection caused by root knot nematode *Meloidogyne incognita*, which is mainly due to less cholesterol levels in root knot nematode eggs controlled by the saponins in plant extracts [40].

Gastrointestinal nematodes are considered as the crucial parasites in ruminants deteriorating the quality dairy products, hence appealing the exploration of natural phytochemicals bearing anthelmintic potential to avoid synthetic chemicals. The extracts of four *Medicago* species (*M. sativa, M. arborea, M. polymorpha, M. polymorpha*) were examined to find *in vitro* anthelminthic potential of 1% saponins that cause a significant reduction (>80%) in nematode egg hatching of gastrointestinal nematodes of dairy donkeys [41]. In another study, the *Medicago* plant extracts enriched with prosapogenins and saponins were tested for *in vitro* anthelmintic activity for sheep gastrointestinal strongyles (GISs) by the egg hatch test. The prosapogenins and saponins obtained from extracts of *M. polymorpha* cultivars Anglona showed strong inhibition on GIS eggs following a concentration-dependent manner [42].

### 4.2. Cytotoxic Effects

The saponins in alfalfa roots extracts (50 *μ*g ml^−1^) induce over 75% cell death in poplar cells following a dose dependent fashion. This reduction in cell viability was mainly due to saponins-mediated induction of nitric oxide (NO) and reactive oxygen species (ROS) production, where the former found quite responsive to sodium azide and N^*G*^monomethyl-L-arginine, which are the specific inhibitors of specific cellular pathways involved in NO biosynthesis in the plant cells isolated from poplar [43]. In another study, brine shrimps (*Artemia Salina*) were treated with extracts of twelve different *Medicago* plant species rich in a range of saponins. But, plant extracts of *M. rigidula* and *M. arabica* showed lethal dose_50_ of 4.6 and 10.1 *µ*g/mL, which depicts structure-activity relationship [19].

The different saponin extracts from *M. arabica* tops and roots showed best cytotoxic activity at the highest concentrations (200 *µ*g/ml) against MCF-7 and HeLa cells using cisplatin as a positive control, and showed only 14 and 23% of cell survival, respectively. In this study, saponins (monodesmosides of hederagenin and bayogenin) rich plant extracts mainly containing 1, 3-*O*-*β*-D-glucopyranosyl (1⟶2)-*α*-L-arabinopyranosyl hederagenin potentially reduced the proliferation of MCF-7 and HeLa cells at 24 hours.

### 4.3. Antioxidant Potential

The extraction studies reported that *Medicago* plants extracts bear strong antioxidant potential. For instance, various parts (roots, stem, leaves) of *M. sativa* plant ethanolic extracts yield various phenolics, flavonoids, and saponins, all of which show higher antioxidant potential [44]. *M. lupulina* is comparatively less studied species, and its crude methanolic extracts showed antioxidant activity with a Trolox^®^ equivalent antioxidant activity (TEAA) and ferric reducing antioxidant power (FRAP) values of 45.4 *µ*mol Trolox/g dw and 0.2 mmol Fe^2+^/g dw through DPPH and FRAP assay [45]. *In vitro* free radical scavenging activity using DPPH assay was performed using various extracts of *M. sativa* seeds, but ethanolic extracts of seeds and seed sprouts showed maximum and ascending radical scavenging activity in a concentration dependent fashion (10, 20, 30, 40, 50, 60, 70, 80, 90, 100 *µ*g) [46].

### 4.4. Antimicrobial Effects

The extracts of *M. sativa* have strong inhibitory effect on *Proteus vulgaris*, *Escherichia coli* (*E. coli*), *Klebsiella pneumonia*, *Salmonella typhi*, *Mucor circinelloides*, *Rhizopus azygosporus*, and *R. microsporus* with less pronounced action on *Shigella flexneri*, *Staphylococcus epidermidis*, *Candida albicans*, and *Emericella quadrillineata* [10]. Moreover, a reversed influence on *Pseudomonas aerugenosa* and *Streptococcus pyrogenes* was seen, while *Pseudallescheria ellipsoidea*, two species of *Penicillium*, and five of *Aspergillus* were seen somewhat resistant for these plant extracts [10].

*M. sativa* plant extracts rich in saponins showed strong antifungal potential to successfully check the growth of *Candida albicans* along with certain clinical pathogenic fungal strains mainly by inhibiting the germ tube formation, retarded the growth of fungal hyphae, and lessened the adherence of yeast cells and eradication of biofilm development at 24 hours after treatment [47]. It is further stated that saponin extracts of *M. sativa* in a dosage range harmful to check the growth of fungi are least toxic to the mice fibroblast L929 cells, which showed them being safe to use for human antifungal conditions [47].

### 4.5. Miscellaneous Effects

The excessive accumulation of ROS at cellular level along with chronic disregulation of cellular antioxidant defense systems leads to diverse pathologies of certain neurodegenerative issues such as Parkinson's disease, amyotrophic lateral sclerosis, Alzheimer's disease, and Huntington disease etc. Li and colleagues isolated three pentacyclic triterpenoid saponins along with medicagenic acid from *M. sativa* using 70% ethanolic extraction and studied their neuroprotective effects using human neuroblastoma SHSY5Y cells [35].

Peroxisome proliferator-activated receptor (PPAR*γ*) is an important regulator of glucose and lipid homeostasis as well as an important pharmacological target for treating metabolic diseases. Saponins and sapogenins found in *Medicago* species showed antagonist potential against PPAR*γ*, which could be helpful to restrain differentiation in adipocytes [24]. Contemporary studies indicate that extracts rich in saponins are effective in lowering blood cholesterol levels. The potential beneficial effects of alfalfa saponins and flavonoids in agriculture and horticulture with regard to protecting plants against pests seem to be of great interest.

### 4.6. Saponins in Dietary Supplements

Various studies reported the use of alfalfa saponins in dietary supplements and are said to be linked with blood plasma parameters, nutrients digestibility, and growth performance of the cattle [48]. *Medicago* species mixed as hay and in silage are considered as significant food for herbivorous fauna, and a rich source of proteins and physically effective neutral detergent fiber for grazers [49]. Within natural grazing systems particularly in meadows, the intake of various classes of compounds like alkaloids, tannins, and saponins is being neutralized to give comfort to the grazers [50].

### 4.7. Bioavailability of the Saponins

The saponins have got permeability barrier across the cellular membranes for their large molecular weights. Hence the bioavailability of saponins should be checked as potential drugs. This major issue with larger molecular structures of saponins rendered them to catch the attention for utilization in drug industry. Recently, huge attempts were made to find the pharmacokinetics potential of these compounds (ginsenosides, astragaloside IV, clematichinenoside AR, and methylprotodioscin) sourced from different plants. In an attempt to find the reasons for the less permeability and reduced bioavailability of saponins, an in silico comparative study was done with crucial physicochemical parameters of cardiotonic drugs sourced from saponins/natural products to elucidate intestinal absorption and bioavailability [51].

## 5. Conclusion

The article summarizes the updates and latest advancements in various biological and pharmacological activities of structurally diverse saponins occurring in the genus *Medicago*. Medicago species (*M. sativa L.*) are being used in traditional medicine systems due to the presence of unique saponins. The article produces the data of chemical structures and molecular masses of all saponins simultaneously. The biological activity of saponins is dependent on the number of side sugar chains attached to the sapogenins as well as to the nature of the sapogenin itself [52]. Monodesmosidic compounds were generally reported to be more biologically active than the corresponding bidesmosidic saponins [19]. For example, when pure aglycones have been used in *in vitro* bioassays, hederagenin was shown to be even more toxic than medicagenic acid and bayogenin against X. *index*, while soyasaponin I, containing soyasapogenol B as a glycone, was the less-active saponin [53]. It is confirmed that structural diversity has a close relationship with its biological and/or pharmacological activities. It is suggested that more sophisticated techniques are needed to isolate more novel saponins for industrial, agricultural, and food manufacturing industries.

## Figures and Tables

**Table 1:** Saponins identified in *M. arabica* leaves.

Aglycone	No.	3-OH substituted	28-COOH substituted	30-CH_3_ substituted	Formula weight	Ref.
*2β*,*3β*,*30-Trihydroxyolean-12-en-28-oic acid*	1^∗^*∗*	*α*-L-Ara(1⟶2)-*β*-D-GluA	—	*β*-D-Glc	C_47_H_74_O_20_ 958	[22]
	2^∗^*∗*	*β*-D-GluA	—	*α*-L-Ara(1⟶2)-*β*-D-Glc	C_47_H_74_O_20_ 958	[22]
	3^∗^*∗*	*β*-D-GluA	—	*β*-D-Glc	C_42_H_66_O_16_ 826	[22]

*3β*,*30-Dihydroxyolean-12-en-28-oic acid*	4^∗^*∗*	*α*-L-Ara(1⟶2)-*β*-D-GluA	—	*β*-D-Glc	C_47_H_74_O_19_ 942	[22]
	5^∗^*∗*	*β*-D-GluA	—	*α*-L-Ara(1⟶2)-*β*-D-Glc	C_47_H_74_O_19_ 942	[22]

*Hederagenin*	6	*α*-L-Ara(1⟶2)-*β*-D-Glc-(1⟶2)-*α*-L-Ara	*β*-D-Glc	—	C_52_H_84_O_22_ 1060	[22]
	7	*β*-D-Glc-(1⟶2)-*α*-L-Ara	*β*-D-Glc	—	C_47_H_76_O_18_ 928	[22]
	8	*α*-L-Ara	*β*-D-Glc	—	C_41_H_66_O_13_ 766	[22, 25]
	9	*α*-L-Ara(1⟶2)-*β*-D-Glc-(1⟶2)-*α*-L-Ara	—	—	C_46_H_74_O_17_ 898	[22]
	10	*β*-D-Glc-(1⟶2)-*α*-L-Ara	—	—	C_41_H_66_O_13_ 766	[6, 22, 25]
	11	*α*-L-Ara(1⟶2)-*β*-D-GluA	—	—	C_41_H_64_O_14_ 780	[22]
	12	*α*-L-Ara	—	—	C_35_H_56_O_8_ 604	[22, 25]

*Bayogenin*	13	*α*-L-Ara	*β*-D-Glc	—	C_41_H_64_O_14_ 782	[22, 25]
	14	*α*-L-Ara	—	—	C_35_H_56_O_9_ 620	[22, 25]

*2β-Hydroxy oleanolic acid*	15	*α*-L-Ara(1⟶2)-*β*-D-GluA	*β*-D-Glc	—	C_47_H_74_O_19_ 942	[22]
	16	*β*-D-GluA	—	—	C_36_H_56_O_10_ 648	[22]

*Soyasapogenol B*	17	L-Rha(1⟶2)-*β*-D-Gal-(1⟶2)-*β*-D-GluA	—	—	C_48_H_78_O_18_ 942	[6, 22]

*Oleanolic acid*	18	*α*-L-Ara(1⟶2)-*β*-D-GluA	—	—	C_41_H_64_O_13_ 764	[22]
	19	*β*-D-GluA	—	—	C_36_H_56_O_9_ 632	[22]

**Table 2:** Saponins from *M. marina* leaves and roots.

Aglycone	No.	3-OH substituted	28-COOH substituted	Formula weight	Ref.
*Zanhic acid*	20^∗^*∗*	*β*-D-Glc(1⟶2)-*β*-D-Glc	*β*-D-Xyl(1⟶4)-[*β*-D-Api(1⟶3)]*-α*-L-Rha(1⟶2)-*α*-L-Ara	C_63_H_100_O_33_ 1384	[26]
	21^∗^*∗*	*β*-D-Glc(1⟶2)-*β*-D-Glc	*β*-D-Xyl(1⟶4)-*α*-L-Rha(1⟶2)-*α*-L-Ara	C_58_H_92_O_29_ 1252	[26]
	22	*β*-D-Glc(1⟶2)-*β*-D-Glc	*β*-D-Xyl(1⟶4)-[*β*-D-Ara(1⟶3)]*-α*-L-Rha(1⟶2)-*α*-L-Ara	C_63_H_100_O_33_ 1384	[26]
	23	*β*-D-Glc	*β*-D-Xyl(1⟶4)-[*β*-D-Ara(1⟶3)]*-α*-L-Rha(1⟶2)-*α*-L-Ara	C_57_H_90_O_28_ 1222	[26]
	24	*β*-D-Glc	*β*-D-Xyl(1⟶4)-*α*-L-Rha(1⟶2)-*α*-L-Ara	C_52_H_82_O_24_ 1090	[26]

*Medicagenic acid*	25^∗^*∗*	*β*-D-Glc(1⟶2)-*β*-D-Glc	*β*-D-Xyl(1⟶4)-[*β*-D-Ara(1⟶3)]*-α*-L-Rha(1⟶2)-*α*-L-Ara	C_63_H_100_O_32_ 1368	[26]
	26^∗^*∗*	*β*-D-Glc	*β*-D-Xyl(1⟶4)-[*β*-D-Ara(1⟶3)]*-α*-L-Rha(1⟶2)-*α*-L-Ara	C_57_H_90_O_27_ 1206	[26]
	27	*β*-D-Glc(1⟶2)-*β*-D-Glc	*β*-D-Xyl(1⟶4)-[*β*-D-Api(1⟶3)]*-α*-L-Rha(1⟶2)-*α*-L-Ara	C_63_H_100_O_32_ 1368	[26]
	28	*β*-D-Glc	*β*-D-Xyl(1⟶4)-*α*-L-Rha(1⟶2)-*α*-L-Ara	C_52_H_82_O_23_ 1074	[26]

*Soyasapogenol B*	29	*α*-L-Rha(1⟶2)-*β*-D-Gal(1⟶2)-*β*-D-GluA	—	C_48_H_78_O_18_ 942	[26]
*Soyasapogenol E*	30	*α*-L-Rha(1⟶2)-*β*-D-Gal(1⟶2)-*β*-D-GluA	—	C_48_H_76_O_18_ 940	[26]

**Table 3:** Saponins from *M. polymorpha* leaves and roots.

Aglycone	No.	3-OH substituted	28-COOH substituted	Formula weight	Ref.
*Echinocystic acid*	31^∗^*∗*	*α*-L-Ara	*β*-D-Glc	C_41_H_66_O_13_ 766	[27]
	32^∗^*∗*	*α*-L-Ara	*β*-D-Glc(1⟶6)-*β*-D-Glc	C_47_H_76_O_18_ 928	[27]
	33	*β*-D-Glc(1⟶2)-*α*-L-Ara	*β*-D-Glc(1⟶6)-*β*-D-Glc	C_53_H_86_O_23_ 1090	[27]
	34	*α*-L-Ara	—	C_35_H_56_O_8_ 604	[27]
	35	*β*-D-Glc	—	C_36_H_58_O_9_ 634	[25]

*Hederagenin*	36	*α*-L-Rha(1⟶2)-*α*-L-Ara	*β*-D-Glc(1⟶6)-*β*-D-Glc	C_53_H_86_O_22_ 1074	[27]
	37	*β*-D-Glc(1⟶2)-*α*-L-Ara	*β*-D-Glc	C_47_H_76_O_18_ 928	[27]
	38	*α*-L-Ara	*β*-D-Glc	C_41_H_66_O_13_ 766	[27]
	39	*α*-L-Rha(1⟶2)-*α*-L-Ara	—	C_41_H_66_O_12_ 750	[25, 27]
	40	*α*-L-Ara	—	C_35_H_56_O_8_ 604	[27]

*Soyasapogenol B*	41	*α*-L-Rha(1⟶2)-*β*-D-Gal(1⟶2)-*β*-D-GluA	—	C_48_H_78_O_18_ 942	[27]

*Caulophyllogenin*	42	*α*-L-Ara	—	C_35_H_56_O_9_ 620	[27]

*Bayogenin*	43	*α*-L-Ara	—	C_35_H_56_O_9_ 620	[27]

**Table 4:** Saponins from *M. truncatula* seeds.

Aglycone	No.	3-OH substituted	28-COOH substituted	Formula weight	Ref.
*Soyasapogenol B*	44	*β*-D-GlcA	*β*-D-Xyl(1⟶4)-*α*-L-Rha(1⟶2)-*α*-L-Ara	C_52_H_84_O_21_ 1044	[28]
	45	*α*-L-Rha(1⟶2)-*β*-D-Gal(1⟶2)-*β*-D-GlcA	—	C_48_H_78_O_18_ 942	[29]

*Medicagenic acid*	46	*β*-D-GlcA	*α*-L-Ara(1⟶2)*-α*-L-Rha(1⟶2)-*β*-D-Xyl	C_52_H_80_O_24_ 1088	[29]
	47	*β*-D-Glc	*α*-L-Ara(1⟶2)*-α*-L-Rha(1⟶2)-*β*-D-Xyl	C_52_H_82_O_23_ 1074	[29]
	48	*β*-D-Glc	*β*-D-Glc	C_42_H_66_O_16_ 826	[29]

**Table 5:** Saponins from *M. sativa.*

Aglycone	No.	3-OH substituted	28-COOH substituted	Formula weight	Ref.
*Medicagenic acid*	49	*β*-D-GlcA	*α*-L-Rha(1⟶2)-*α*-L-Ara	C_47_H_72_O_20_ 956	[30]
	50	*α*-L-Rha(1⟶2)-*β*-D-Gal(1⟶2)-*β*-D-GluA	—	C_48_H_74_O_21_ 986	[31]
	51	*β*-D-GlcA	*β*-D-Xyl(1⟶4)-*α*-L-Rha(1⟶2)-*α*-L-Ara	C_52_H_80_O_24_ 1088	[18, 31]
	52	*β*-D-GlcA	*β*-D-GlcA	C_42_H_62_O_18_ 854	[32]
	53	*β*-D-Glc(1⟶3)-*β*-D-Glc	*α*-L-Rha(1⟶2)-*α*-L-Ara	C_53_H_84_O_24_ 1104	[32]
	54	*β*-D-GlcA	*β*-D-Api(1⟶3)-[*β*-D-Xyl(1⟶4)]-*α*-L-Rha(1⟶2)-*α*-L-Ara	C_57_H_88_O_28_ 1220	[32]
	55	*β*-D-Glc	*β*-D-Glc(1⟶4)-*α*-L-Rha(1⟶2)-*α*-L-Ara	C_53_H_84_O_24_ 1104	[33]
	56	*β*-D-Glc(1⟶2)-*β*-D-Glc	*β*-D-Xyl(1⟶4)-*α*-L-Rha(1⟶2)-*α*-L-Ara	C_58_H_92_O_28_ 1236	[18, 33]
	57	*α*-L-Ara(1⟶2)-*β*-D-Glc(1⟶2)-*α*-L-Ara	*β*-D-Glc	C_52_H_82_O_24_ 1090	[33]
	58	*β*-D-Glc	—	C_36_H_56_O_11_ 664	[1, 18, 25]
	59	*β*-D-Glc	*β*-D-Glc	C_42_H_66_O_16_ 826	[1, 18, 25, 34]
	60	*β*-D-Glc	*β*-D-Xyl(1⟶4)-*α*-L-Rha(1⟶2)-*α*-L-Ara	C_52_H_82_O_23_ 1074	[1, 6, 18]
	61	*β*-D-Glc(1⟶2)-*β*-D-Glc	*β*-D-Glc	C_48_H_76_O_21_ 988	[18, 25]
	62^*∗*^	*β*-D-Glc(1⟶2)-*β*-D-Glc(1⟶2)-*β*-D-Glc	—	C_48_H_76_O_21_ 988	[34]
	63	*α*-L-Rha(1⟶2)*-β*-D-Glc(1⟶2)-*β*-D-Glc	—	C_48_H_76_O_20_ 972	[34]

*Zanhic acid*	64	*β*-D-GlcA	*β*-D-Xyl(1⟶4)-*α*-L-Rha(1⟶2)-*α*-L-Ara	C_52_H_80_O_25_ 1104	[30]
	65	*β*-D-Xyl(1⟶4)-*α*-L-Rha(1⟶2)-*α*-L-Ara	—	C_46_H_72_O_19_ 928	[32]
	66	*β*-D-Glc	*β*-D-Xyl(1⟶4)-*α*-L-Rha(1⟶2)-*α*-L-Ara	C_52_H_82_O_24_ 1090	[32]
	67	*β*-D-Glc(1⟶3)-*β*-D-Glc	*α*-L-Rha(1⟶2)-*α*-L-Ara	C_53_H_84_O_25_ 1120	[32]
	68	*β*-D-Glc	*α*-L-Ara(1⟶3)-[*β*-D-Xyl(1⟶4)]-*α*-L-Rha(1⟶2)-*α*-L-Ara	C_57_H_90_O_28_ 1222	[32]
	69	*β*-D-GlcA	*α*-L-Ara(1⟶3)-[*β*-D-Xyl(1⟶4)]-*α*-L-Rha(1⟶2)-*α*-L-Ara	C_57_H_88_O_29_ 1236	[32]
	70	*β*-D-Glc(1⟶3)-*β*-D-Glc	*α*-L-Ara(1⟶3)-*α*-L-Rha(1⟶2)-*α*-L-Ara	C_58_H_92_O_29_ 1252	[32]
	71	*β*-D-Glc(1⟶2)-*β*-D-Glc	*α*-L-Ara(1⟶3)-[*β*-D-Xyl(1⟶4)]-*α*-L-Rha(1⟶2)-*α*-L-Ara	C_63_H_100_O_33_ 1384	[32]
	72	*α*-L-Ara(1⟶2)-*β*-D-Glc(1⟶2)-*β*-D-Glc	*β*-D-Api(1⟶3)-[*β*-D-Xyl(1⟶4)]-*α*-L-Rha(1⟶2)-*α*-L-Ara	C_68_H_108_O_37_ 1516	[32]
	73	*β*-D-Glc(1⟶2)-*β*-D-Glc(1⟶2)-*β*-D-Glc	*β*-D-Xyl(1⟶4)-*α*-L-Rha(1⟶2)-*α*-L-Ara	C_64_H_102_O_34_ 1414	[1, 18, 25]
	74	*β*-D-Glc(1⟶2)-*β*-D-Glc(1⟶2)-*β*-D-Glc	*β*-D-Api(1⟶3)-[*β*-D-Xyl(1⟶4)]-*α*-L-Rha(1⟶2)-*α*-L-Ara	C_69_H_110_O_38_ 1546	[18, 30]
	75	*β*-D-GluA	*α*-L-Ara(1⟶3)-[*β*-D-Xyl(1⟶4)]-*α*-L-Rha(1⟶2)-*α*-L-Ara	C_57_H_88_O_29_ 1236	[1]

*Bayogenin*	76	*α*-L-Ara	*β*-D-Glc	C_41_H_66_O_14_ 782	[32]
	77^∗^*∗*	*β*-D-Gal(1⟶2)-*β*-D-GluAME	*β*-D-Glu	C_49_H_76_O_22_ 1016	[35]
	78^∗^*∗*	*β*-D-Xyl(1⟶4)-*β*-D-GluAME	*β*-D-Glc	C_48_H_76_O_20_ 972	[35]

*Hederagenin*	79	—	*β*-D-Glc	C_36_H_58_O_9_ 634	[32]
	80	*α*-L-Ara(1⟶2)-*β*-D-Glc	*β*-D-Glc	C_47_H_76_O_18_ 928	[18]
	81	*α*-L-Ara(1⟶2)-*β*-D-Glc(1⟶2)-*α*-L-Ara	—	C_46_H_74_O_17_ 898	[18]
	82	*β*-D-Glc(1⟶2)-*α*-L-Ara	—	C_41_H_66_O_13_ 766	[1]
	83	*α*-L-Ara(1⟶2)-*β*-D-Glc(1⟶2)-*α*-L-Ara	*β*-D-Glc	C_52_H_84_O_22_ 1060	[18, 34]
	84^∗^*∗*	*β*-D-Xyl(1⟶3)-*β*-D-Glc	*β*-D-Xyl(1⟶4)-*α*-L-Rha(1⟶2)-*α*-L-Ara	C_57_H_92_O_25_ 1176	[34]
	85^∗^*∗*	*α*-L-Ara(1⟶2)-*β*-D-Glc(1⟶2)-*β*-D-Xyl	*β*-D-Glc	C_52_H_84_O_22_ 1060	[34]
	86^∗^*∗*	*β*-D-Xyl(1⟶2)-*β*-D-Glc(1⟶2)-*β*-D-Glc	*β*-D-Glc	C_53_H_86_O_23_ 1090	[34]
	87^∗^*∗*	*β*-D-Glc(1⟶2)-*β*-D-Glc(1⟶2)-*α*-L-Ara	*β*-D-Glc	C_53_H_86_O_23_ 1090	[34]
	88^∗^*∗*	*α*-L-Ara(1⟶2)-*β*-D-Glc(1⟶2)-*β*-D-GluAME	*β*-D-Glc	C_54_H_86_O_24_ 1118	[35]
	89	*β*-D-Glc(1⟶2)-*α*-L-Ara	*β*-D-Glc	C_47_H_76_O_18_ 928	[34]

*2β*,*3β-Dihydroxy-23-oxo-olean-12en-28oic acid*	90	*β*-D-GlcA	*β*-D-Glc	C_42_H_64_O_16_ 824	[32]
	91^∗^*∗*	*β*-D-Xyl(1⟶2)-*β*-D-Glc(1⟶2)-*β*-D-Glc	*β*-D-Glc	C_53_H_84_O_24_ 1104	[34]

*Soyasapogenol B*	92	*α*-L-Rha(1⟶2)-*β*-D-Gal(1⟶2)-*β*-D-GluA	—	C_48_H_78_O_18_ 942	[1, 18, 25, 33, 34]

**Table 6:** Saponins from *M. arborea.*

Aglycone	No.	3-OH substituted	28-COOH substituted	Formula weight	Ref.
*Medicagenic acid*	93	*β*-D-GlcA	*β*-D-Xyl(1⟶4)-*α*-L-Rha(1⟶2)-*α*-L-Ara	C_52_H_80_O_24_ 1088	[1, 25]
	94	*β*-D-Glc	*β*-D-Xyl(1⟶4)-*α*-L-Rha(1⟶2)-*α*-L-Ara	C_52_H_82_O_23_ 1074	[1, 25]
	95	*β*-D-Glc	—	C_36_H_56_O_11_ 664	[1]

## Data Availability

All data used to support the findings of this study are included within the paper.
